# Comparison of CMIP6 GCMs historical precipitation with measured precipitation over Pakistan

**DOI:** 10.1371/journal.pone.0319999

**Published:** 2025-03-31

**Authors:** Adnan Abbas, Waheed Ullah, Safi Ullah, Asher Samuel Bhatti, Muhammad Waseem, Gohar Ali, Dayong Xu

**Affiliations:** 1 School of Chemical and Environmental Engineering, Anhui Polytechnic University, Wuhu, Anhui, China; 2 Defense and Security, Rabdan Academy, Abu Dhabi, United Arab Emirates; 3 Department of Atmospheric and Oceanic Sciences, Fudan University, Shanghai, China; 4 Department of Geology, Bacha Khan University, Charsadda, Pakistan; 5 Center of Excellence in Water Resources, University of Engineering and Technology, Lahore, Pakistan; 6 Pakistan Meteorological Department, Sector H-8/2, Islamabad, Pakistan; Euro-Mediterranean Center for Climate Change: Fondazione Centro Euro-Mediterraneo sui Cambiamenti Climatici, ITALY

## Abstract

Comparison of Coupled Model Intercomparison Project Phase 6 (CMIP6) General Circulation Models (GCMs) with observations under different climatic conditions is necessary to determine their respective strengths and differences. In the current study, ten CMIP6 GCMs are compared with measured gauge precipitation data of 51 stations across Pakistan. Results show reasonable agreement between the CMIP6-GCMs with measured data in capturing precipitation days of ≤10 mm/day. The precipitation intensity of events ≥10 mm/day shows a significant resemblance with measured data at a 95% confidence level (K-S test). Furthermore, the results of regional differences demonstrate the relatively good agreement of CMCC-CM2-SR5, EC-Earth3-AerChem, and EC-Earth3-CC with measured data in arid and semiarid regions and FGOALS-f3-L in humid and extremely arid regions. Significant precipitation variability is reported in the interannual standard deviation ratio (STD) for all GCMs in all seasons, implying more dynamics and intense precipitation in GCMs. The magnitude of STD is sensitive to the precipitation magnitude in time and space rather than climate classes, higher and lower in monsoon and autumn seasons, respectively. The climatological mean shows higher precipitation in the northeastern and southeastern parts of GCMs during the monsoon and lower precipitation during winters complementing station data. Based on selected metrics, CMCC-ESM2 has the highest skill in simulating precipitation distributions over Pakistan, followed by CMCC-CM2-SR5 and EC-Earth3-CC, while NorCPM1 ranked the worst in reproducing measured precipitation. The findings can serve as a benchmark in the region for applying the CMIP6 GCMs in water and food security studies.

## 1. Introduction

Climate change will likely impact the regional water and energy cycle, profoundly challenging arid regions’ water and food security [1,2]. The regions with abrupt changes and subtle variations in climatic variables (precipitation in particular) are more vulnerable to the consequences of climate change [3,4]. Pakistan has ranked among the top ten climate-change-impacted regions due to its exposure to climate extremes and post-extreme hazards [5]. Hence, analyzing historical extreme precipitation events in Pakistan is of utmost significance for policymakers to account for regional sustainable development, food security, and population exposure to hazards [2]. Such an analysis of the existing remotely sensed precipitation products, reanalysis, and gauge precipitation data has offered valuable insights [6,7]; less attention is given to the historical and future extreme events. Furthermore, the spatiotemporal changes in precipitation frequency and intensity adversely impact agricultural production, water resources, energy, and power generation, and the environment, prompting the need for more studies for a better understanding [8–10].

The complex morphology of precipitation makes it challenging to predict and mitigate its severe socio‐economic impacts [11,12]. Global climate models (GCMs) can analyze the spatial impact of greenhouse gas emissions on climate change and reveal the historical changes in precipitation patterns. The major advantage of GCMs is using an integrative system (land, atmosphere, and ocean interactions) to evaluate the long-term changes in climatic patterns [13,14]. The Coupled Model Intercomparison Projects (CMIP) released a new set of GCMs in its 6th phase [15]. The historical simulations of CMIP6 GCMs used volcanic eruptions and solar variability as natural drivers, and CO_2_ concentration, aerosols, and land use as human-induced factors for climate change interventions [16]. CMIP6 GCMs are coupled with additional physical complexity and a higher spatial resolution than the previous phase, i.e., CMIP5 [17,18]. The historical simulations from CMIP6-GCMs are the “entry card” for all models participating in the CMIP6 project [16]. Akinsanola and Zhou [19], and Barlow et al. [20] claimed that the additional improvements in CMIP6 may not necessarily improve model performance in simulating the precipitation characteristics on a regional scale. Su et al. [21] also illustrated fluctuations within CMIP6 GCM outputs relative to observations over different regions. Given these enhancements, CMIP6 offers several improvements over CMIP5, including higher resolution, updated physical parameterizations, and new forcing datasets like aerosols and land-use changes. These advancements enhance the models’ ability to simulate precipitation patterns, particularly in complex regions like Pakistan [6,22], where an accurate representation of monsoonal and extreme precipitation is crucial. These improvements are central to our assessment of GCM performance in simulating historical precipitation under varying climatic conditions. Thus, it is essential to study and evaluate the performance of CMIP6 GCMs over different subcontinents.

GCMs are generally used to investigate the changes in spatial extent, time, duration, frequency, and intensity of precipitation [23]. However, biases in precipitation intensity and frequency in GCMs have been reported in recent studies [24–26] that warrant thorough evaluations before their applications [27,28]. Thus, it is imperative to evaluate their performance (GCMs) relative to measured data or independent precipitation data for impact applications and policy-making [29–31]. Hence, in the current study, we evaluate CMIP6 GCMs to simulate seasonal precipitation over Pakistan’s diverse climatic and topographic regions. Pakistan is among the vulnerable regions to climate change due to its geographical location, low coping capacity, dynamic climatology, and complex topography [6,7,32]. During the evaluation period (1981–2014), Pakistan experienced several notable hydrometeorological disasters driven by dynamic changes in precipitation extremes. For instance, the devastating floods of 2010 were among the worst in the country’s history, affecting over 20 million people and causing widespread infrastructure damage. Moreover, the 1992 floods were triggered by heavy monsoon rains and glacial melt, affecting large parts of Punjab and Khyber Pakhtunkhwa, resulting in widespread destruction and loss of life. Another significant event was the drought of 1998–2002, which severely impacted agriculture, leading to water shortages and economic losses, particularly in the southern regions. These examples illustrate the scale of hydrometeorological disasters in Pakistan during the evaluation period, highlighting the importance of understanding precipitation dynamics in future projections [33,34]. These events underscore the critical role of extreme precipitation in causing hydro-meteorological disasters in Pakistan, as highlighted in the study [35–37].

Several studies have been conducted to evaluate the performance of CMIP-GCMs over time to assess the changes in the regional water and energy cycle. For instance, Ahmed et al. [38] evaluated CMIP5-GCMs in simulating historical precipitation and temperature over Pakistan from 1961–2005. They reported that NorESM1-M, MIROC5, BCC-CSM1-1, and ACCESS1-3 are the most skillful GCMs in replicating precipitation and temperature. Iqbal et al. [39] used the Support Vector Machine Recursive Feature Elimination algorithm to evaluate and select suitable CMIP5 GCMs in simulating projected changes in precipitation using the Asian Precipitation - Highly-Resolved Observational Data Integration Towards Evaluation as reference data. They found MIROC5, EC-EARTH, CNRM-CM5, BCC-CSM1.1(m), and BCC-CSM1.1 to be the best-performing models in projecting climate change. From relative assessment, Khan et al. [40] identified 6 CMIP5-GCMs for reliable temperature and precipitation projection over Pakistan, namely ACCESS1-3, CESM1-BGC, CMCC-CM HadGEM2-CC, HadGEM2-ES, and MIROC5. In another study, Latif et al. [41] evaluated the CMIP5 GCMs for seasonal rainfall variability during 1951–2005 over the Indo-Pakistan subcontinent. They found that the HadGEM2-AO and CNRM-CM5 models have successfully reproduced the rainfall climatology and statistical trend metrics.

It should be noted that previous studies have evaluated the performance of CMIP5 models in the simulation of historical precipitation; however, few studies have been conducted to assess the spatiotemporal skills of CMIP6 GCMs in simulating historical precipitation extremes over Pakistan. The current study has thus attempted to compare the available CMIP6 GCMs with gauge precipitation data as a reference in simulating the regional precipitation during 1981–2014. The gauge-measured precipitation represents point data with retrieval and measurement approaches different from GCMs [42,43]. Thus, we acknowledge that this may affect the quantitative and statistical outputs to some degree, but both will preserve the natural patterns of precipitation variability.

## 2. Study area

**Fig 1:**
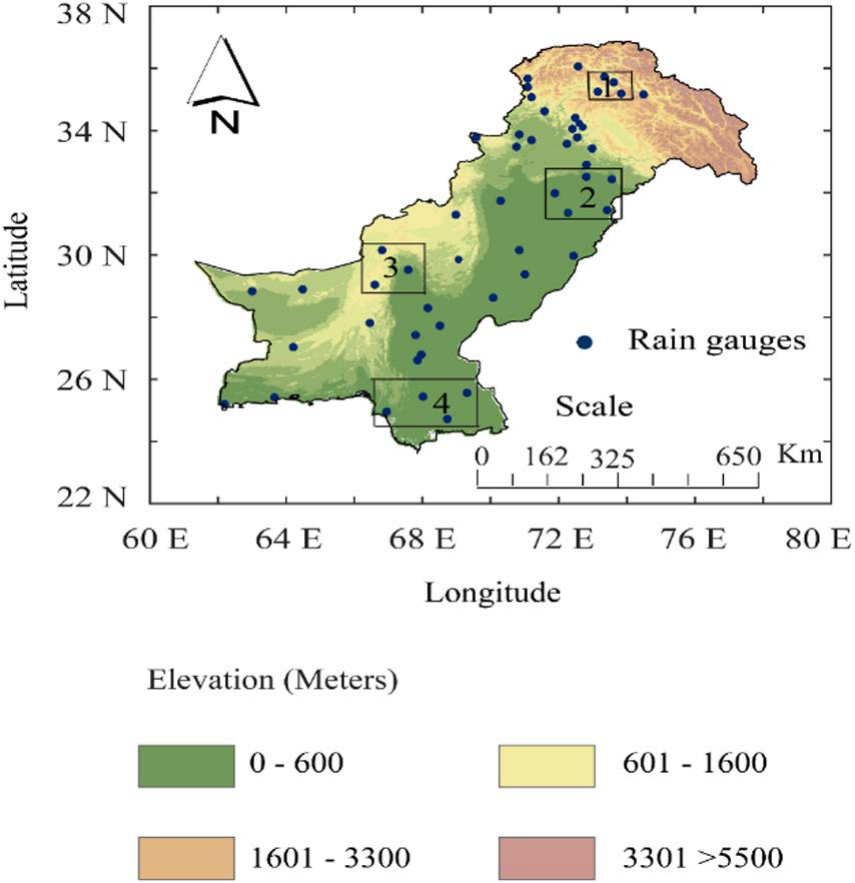
Topography and station density of the study area. The stations (black box) were selected to cover the different climatic regions (i.e., R1, R2, R3, and R4) of Pakistan. The map in Fig 1 was created using publicly available DEM data from NASA’s SRTM and visualized using open-source QGIS software. NASA JPL (Jet Propulsion Laboratory) 2013. https://doi.org/10.5067/MEaSUREs/SRTM/SRTMGL1.003.

Pakistan (Fig 1) is dominantly arid to semiarid in the southern and central plains and relatively humid in the northern mountains [44]. The land cover varies from grassland to barren land, and the climate region varies from arid to humid (Table 1). The topography, temperature, and precipitation closely follow a similar variability pattern from south to north [6,45]. The maximum precipitation and temperature values are experienced during the summer monsoon season [45,46]. Winter and pre-monsoon seasons experience moderate precipitation and lower temperatures due to westerlies and mid-latitude weather fronts [47,48]. This study assesses the performance of Global Circulation Models (GCMs) across Pakistan, focusing on four distinct regions (R1, R2, R3, and R4) characterized by varying climatic conditions and land cover types (Table 1). These regions were delineated based on previous studies that considered elevation, climate classification, and land cover characteristics [7,45]. This approach allows for a comprehensive analysis of GCMs performance in simulating precipitation patterns. While the entire country serves as the broader study area, our detailed analysis centers on these four regions to capture critical climatic zones relevant to water resource and food security research.

**Table 1:** Description of the regional elevation, climate class, and land cover of the four regions with their latitude-longitude ranges.

Regions (R1-R4)	Elevation (m)	Climate	Land cover	Latitude (°)	Longitude (°)
1	3001–6000	Semi-arid	Grassland	34–36	72–74
2	0–600	Humid	Cropland	30–34	70–74
3	601–1600	Arid	Shrubland	28–30	66–68
4	0–600	Extremely Arid	Cropland	24–26	66–70

## 3. Data and methods

### 3.1. Data

This reference daily precipitation data from 1981-2014 is obtained from the Pakistan Meteorological Department (PMD) for 51 stations (Fig 1). The PMD operates a dense network of the rain-gauges across the country including telemetric and automatic observations as well as manual rain-gauges. The type of rain-gauge installed regionally varies, which depends on the location, accessibility, and historical setup of the station. Over the years regular updates to the type of rain-gauge can impact the measurement sensitivity, recording intervals and potential biases from underestimation of tipping-bucket rain-gauges, sampling error, wind and calibration errors [43], which can influence the cumulative distribution [49,50]. The PMD processes the data collected on multiple scales for different applications using the WMO (CIMO, 2014) [51]. We further checked the data for inconsistency including environmental and non-environmental factors using the Standard Normal Homogeneity Test (SNHT) [45,49].

For CMIP6 GCMs, the available daily precipitation data of ten (10) models listed in Table 2 are obtained from the CMIP6 data archives (https://esgf-node.llnl.gov/search/cmip6/) for the historical period of 1981–2014. These ten models are selected since they can simulate many climate variables from different model configurations to account for the dynamics of the atmosphere, ocean, sea-ice, and land components. Indeed, there are more than 35 GCMs in the CMIP6 data archive, which have precipitation outputs. So far, several studies have assessed the performance of most of those CGMs against different gridded products (i.e., CRU, ERA5, GPCC, etc.) over Asia, South Asia, and Pakistan with different intended application [1,40,52]. In this study we selected these models considering a diverse characteristic to better assess the robustness of precipitation. The model selection considers diversity with intermediate resolution and physical parameterization of aerosol-cloud-precipitation interactions, carbon cycle feedback, different data assimilation approaches, regional simulation abilities, and model independence, which distinguish them. We also consider the computational constraints for selecting subsets of models to efficiently capture a diverse range of model behaviors, incorporating models that complement previous studies while including some models that were not previously assessed. Furthermore, only the first available ensemble member of each model is selected in the current study. The selected GCMs are further interpolated into a common grid of 0.5o × 0.5o resolution using bilinear interpolation. The details of the selected CMIP6 GCMs are provided in Table 2.

**Table 2:** A detailed description of the CMIP6 GCMs used in this study.

Sr. No.	Models	Institution	Horizontal resolution (Lon-Lat)
1	BCC-CSM2-MR	Beijing Climate Center, China	1.1° × 1.1°
2	CESM2-WACCM	National Center for Atmospheric Research (NCAR), USA	1.3° × 0.9°
3	CMCC-CM2-SR5	Fondazione Centro Euro-Mediterraneo sui Cambiamenti Climatici, Italy	1.3° × 0.9°
4	CMCC-ESM2	Fondazione Centro Euro-Mediterraneo sui Cambiamenti Climatici, Lecce 73100, Italy	1.3° × 0.9°
5	EC-Earth3-AerChem	EC-Earth-Consortium, Europe	0.7° × 0.7°
6	EC-Earth3-CC	EC-Earth-Consortium, Europe	0.7° × 0.7°
7	FGOALS-f3-L	Chinese Academy of Sciences, China	1.3° × 1.0°
8	MPI-ESM-1-2-HAM	ETH Zurich, Switzerland; Max Planck Institut fur Meteorologie, Germany; Forschungszentrum Julich, Germany; University of Oxford, UK; Finnish Meteorological Institute, Finland; Leibniz Institute for Tropospheric Research, Germany; Center for Climate Systems Modeling (C2SM) at ETH Zurich, Switzerland	1.5° × 1.5°
9	MRI-ESM2-0	Meteorological Research Institute (MRI), Japan	1.1° × 1.1°
10	NorCPM1	NorESM Climate Modeling Consortium (NCC), Norway	1.0° × 1.0°

### 3.2. Methods

The performance evaluation of the selected GCMs is carried out on different temporal (daily, interannual, and seasonal) scales and spatial scales. For temporal comparison, the regional precipitation data from the stations in each region (Table 1) are averaged and compared with the GCMs. The regional variability of precipitation is the primary factor in defining the climate regions accompanied by climate class, land cover, and terrain attributes. The GCM precipitation is retrieved from the nearest pixel to the station’s location and averaged for each region. For spatial comparison, a similar approach is used to compare the precipitation of the nearest pixel to the station’s location for each GCM. Furthermore, frequency versus intensity plots and Empirical Cumulative Distribution Function (ECDF) are used to compare the CMIP6 GCMs with station data for daily scale comparison. For station data and CMIP6-GCMs, a precipitating day is defined when the total daily precipitation recorded is > 0.25 mm/day [45]. Due to high precipitation variability in time and space, we use the ECDF and frequency vs intensity histograms (bin size = 1.75 mm) to quantify the cumulative precipitation frequency and intensity distribution in different bins.

For seasonal scale evaluation, the temporal series of precipitations are classified into four seasons such as winter (December, January, February, and March; DJFM), pre-monsoon (April, May, and June; AMJ), monsoon (July, August, and September; JAS), and autumn (October and November; ON) following [45]. Moreover, historical evaluation of CMIP6 GCMs in simulating the seasonal extremes over different climatic regions of Pakistan, ≥1 mm/day (wet days), ≥2.5 mm/day (moderate precipitation days), ≥5 mm/day (heavy precipitation days), and ≥10 mm/day (very heavy precipitation days) precipitation intensity thresholds are used [53]. A non-parametric Kolmogorov-Smirnov (K-S) significance test with a 95% confidence level is applied to compare the similarity of precipitation between the station data and GCMs.

For interannual scale comparison, the standard deviation ratio between the measured data and the CMIP6 GCMs is used to show the relative performance of the GCMs in capturing the precipitation pattern variability. Furthermore, the probability density function (PDF) is used at the annual scale to observe the relative density of precipitation from GCMs relative to the measured data. The PDF is a non-parametric method using kernel density estimation (KDE) on a random sample. A kernel usually describes the distribution pattern allowing for the input shaping the distribution pattern and moments of distribution such as mean, kurtosis, standard deviation, and skewness.

Additionally, four error metrics, i.e., bias, mean absolute difference (MAD), the ratio of standard deviation (STD), and *unbiased* root mean–square difference (*ub*RMSD), are used to rank the GCMs based on their simulations with measured data [54]. These metrics are interdependent on each other. Among these metrics, bias, and MAD are the measures of differences within the mean and amplitude of measured and GCMs, the STD provides a statistical summary of the ratio of amplitudes, and *ub*RMSD reports the differences [45,55]. The mathematical formulas of bias, MAD, STD, and *ub*RMSD are expressed in Equations (1), (2), and (3), respectively.


$$Bias=\frac{1}{n}\overset{n}{\underset{i=1}{\sum }}\frac{\left(GCM_{i}-ME_{i}\right)}{ME_{i}}$$ (1)

$$MAD=\frac{1}{n}\overset{n}{\underset{i=1}{\sum }}\left|GCM-ME\right|$$ (2)

$$ubRMSD=\sqrt{\frac{\sum_{i=1}^{n}[(ME_{i}-\bar{ME_{i})}-(GCM_{i}-{\bar{GCM_{i}]}}^{2}}{n}}$$ (3)

$$STD=\frac{\sigma_{GCM}}{\sigma_{ME}}$$ (4)

Where *ME* and *GCM* represent the precipitation product from measured and CMIP6 GCM datasets, respectively, n is the number of observations of the respective time period *i* under consideration, and σ is the standard deviation.

## 4. Results

### 4.1. Daily variability of precipitation

**Fig 2:**
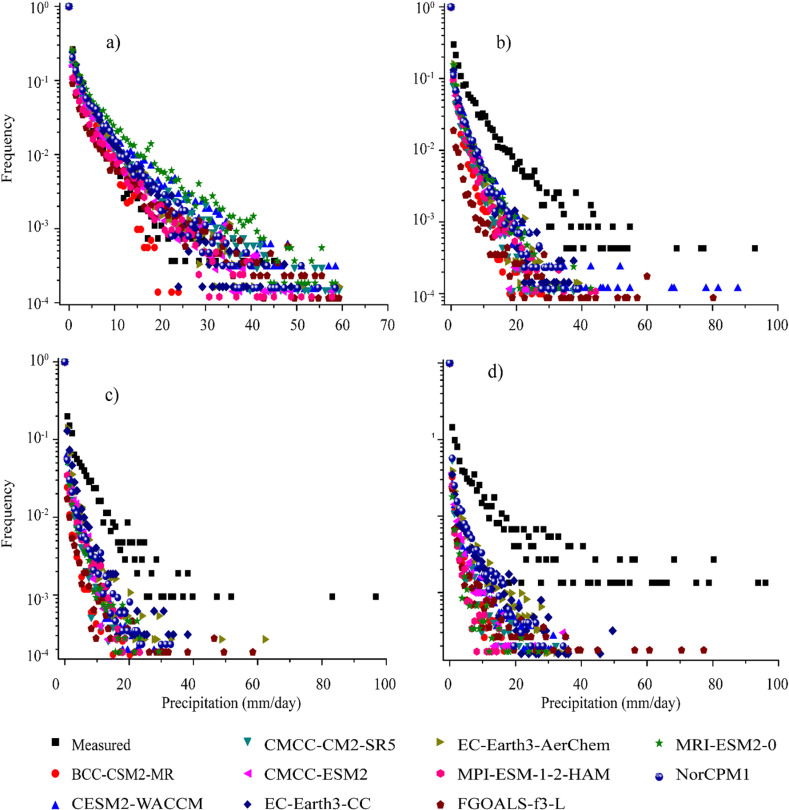
Normalized frequency of daily precipitation (bin size: 1.75 mm) in different climatic zones for the measured data and GCMs; **(a)** R1, **(b)** R2. **(c)** R3, and **(d)** R4.

Fig 2 shows the frequency and intensity histograms of the daily precipitation distribution with a bin size of 1.75 mm in four climatic regions, i.e., R1, R2, R3, and R4. A precipitation day with less than 0.25 mm is considered a trace and excluded from both the GCM and station data analyses. Meanwhile, the 0 to 1.75 mm/day range refers to light precipitation events above the trace threshold [45,56]. Fig 2a shows a relatively lower frequency of precipitating days and precipitation amount in the station data than the CMIP6 GCMs, illustrating an overestimation of precipitation by all CMIP6 GCMs in R1. The precipitation spread infers a uniform precipitation frequency and intensity up to 30 mm/day and a divergence afterward between the station data and all GCMs. In Fig 2b (R2), both the station data and the CMIP6 GCMs have more precipitating days below 20 mm/day and fewer precipitating days with > 30 mm/day precipitation. The precipitation pattern in CMIP6 GCMs shows an underestimation compared to station precipitation data. In R3 and R4 (Fig 2c and d), a similar pattern of the precipitation difference between the station data and the CMIP6 GCMs shows more precipitation days under 30 mm/days and fewer precipitating days > 30 mm/day. The CMIP6 GCMs in simulating lower precipitating days agree and diverge for higher precipitating days compared to the station data. With increasing precipitation ≥10 mm/day, all the selected GCMs tend to underestimate the frequency of precipitation events in R2, R3, and R4. CMCC-ESM2, FGOALS-f3-L, and MPI-ESM-1-2-HAM perform relatively better in capturing the frequency of precipitation events. Moreover, MRI-ESM2-0, NorCPMI, and CESM2-WACCM exhibit a high frequency of precipitation events in R2. At the same time, Fig 2b and d reveal that EC-Earth3-AerChem and NorCPMI demonstrate a relatively better precipitation event frequency concerning the station data.

Conclusively, Fig 2 illustrates the maximum frequency of the wet days in the first bin (0 to 1.75 mm/day) equally reported by all GCMs. The frequency of precipitating days further shows that CMIP6 GCMs generally performed well in exhibiting the frequency and intensity of precipitation events at R1; however, for the rest of the regions (i.e., R2, R3, and R4), deviations and differences are observed in capturing precipitation frequencies relative to the station precipitation data.

**Fig 3:**
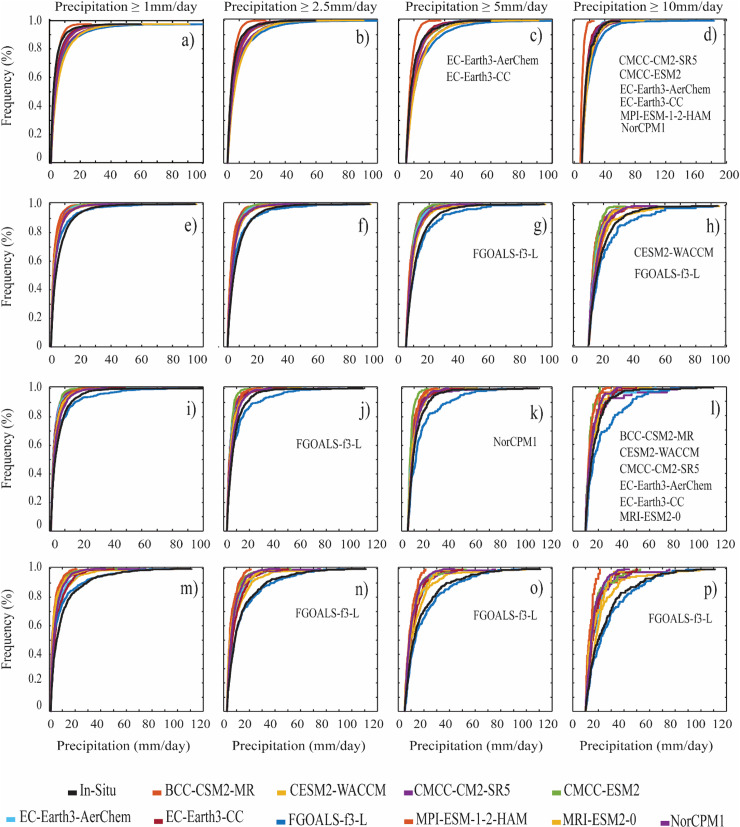
Comparisons of the empirical cumulative distribution functions (ECDFs) of daily precipitation indices for the measured data and GCMs; (a– **d)** R1, (e–**h)** R2), (i–**l)** R3, and (m–**p)** R4.

The performance of the CMIP6 GCMs against the measured data is further evaluated using ECDFs on a daily scale with four precipitation indices, i.e., ≥1 mm/day, ≥2.5 mm/day, ≥5 mm/day, and ≥10 mm/day in the selected four regions. Non-parametric K-S test (95% confidence bound) identifies the CMIP6 GCMs that significantly matched the station precipitation distribution. Fig 3 shows the results of the CMIP6 GCMs in capturing the empirical cumulative distribution of precipitation events relative to the station data. The CMIP6 GCMs show significant similarity with the station data named inside their respective threshold boxes. Fig 3a–d shows that the precipitation ECDF from all CMIP6 GCMs closely reflects the station precipitation distribution with a mixed pattern of slightly overestimation and underestimation in R1. The skills of CMIP6 GCMs in capturing higher (Fig 3c and d) precipitation events are higher than smaller precipitation events. For precipitation events characterized by ≥10 mm/day, CMCC-CM2-SR5, CMCC-ESM2, EC-Earth3-AerChem, EC-Earth3-CC, MPI-ESM-1-2-HAM, and NorCPMI significantly demonstrated a similar pattern to that of the measured data.

Fig 3e–h shows that ≥1 mm/day and ≥2.5 mm/day events are underestimated except FGOALS-f3-L, which resembles the station precipitation distribution in R2. Except for CESM2-WACCM, the precipitation is overestimated for higher events of ≥5 mm/day and ≥10 mm/day by the selected GCMs. For precipitation events of ≥10 mm/day, all the CMIP6 GCMs underestimate the observed precipitation, while FGOALS-f3-L and CESM2-WACCM show significantly closer agreement with that of measured precipitation compared to the other GCMs, although the observed precipitation is overestimated in FGOALS-f3-L. In R3 (Fig 3i–l), all the CMIP6 GCMs underestimate the precipitation frequency of the measured data except FGOALS-f3-L. Irrespectively, no significant resemblance between the measured data and CMIP6 GCMs is observed for precipitation threshold ≥1 mm/day.

For the precipitation threshold ≥2.5 mm/day, FGOALS-f3-L significantly produces the ECDF, though the precipitation is overestimated, while NorCPMI has significant similarity with the measured data for the precipitation threshold ≥5 mm/day. The performance of CMIP6 GCMs significantly improves for the precipitation threshold ≥10 mm/day with BCC-CSM2-MR, CESM2-WACCN, CMCC-CM2-SR5, EC-Earth3-AerChem, EC-Earth3-CC, and MRI-ESM2-0 being able to produce ECDF significantly. In R4 (Fig 3m–p), all the CMIP6 GCMs underestimate the observed precipitation, while FGOALS-f3-L demonstrates a significant similarity to the measured precipitation distribution for ≥2.5 mm/day, 5 mm/day, and ≥10 mm/day precipitation events.

In conclusion, the daily scale precipitation frequencies and extreme precipitation events are reasonably captured by the CMIP6 GCMs with a significant improvement in the model performance with an increase in the daily precipitation. Except for R1, the smaller precipitation events are underestimated, and a significantly similar distribution with the measured data is observed for higher precipitation magnitudes. CMCC-CM2-SR5, EC-Earth3-AerChem, and EC-Earth3-CC show relatively significant resemblance in R1 and R3, while FGOALS-f3-L outperforms other models in producing similar precipitation distribution in R2 and R4. It is worth mentioning that the FGOALS-f3-L model performs better in R2, R3, and R4 for precipitation events ≥2.5 mm/day and ≥5 mm/day. The overall performance suggests that even with a significant overestimation/underestimation and spread of the daily precipitation events, some of the selected GCMs still can capture the frequency and intensity of the measured precipitation, particularly for higher events (i.e., ≥10 mm/day) in R1, R2, and R3. These results are in line with the findings of recent studies [31,57].

### 4.2. Interannual variability

**Fig 4:**
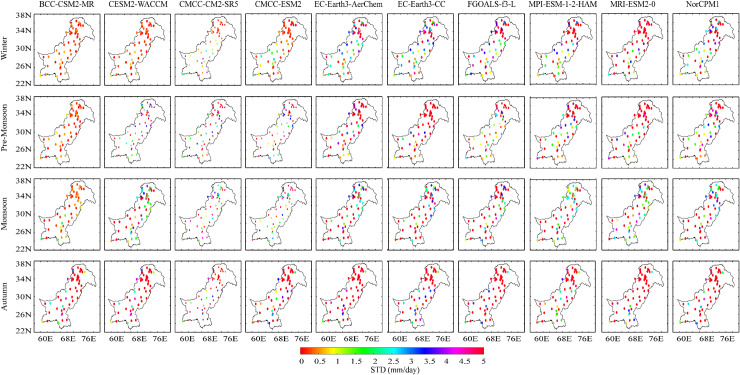
Standard deviation ratio of interannual precipitation of the GCMs and measured data over Pakistan for winter, pre-monsoon, monsoon, and autumn seasons.

Fig 4 shows the interannual standard deviation (STD) ratio for different seasons between the observation and GCMs during 1981–2014. During the winter season, the overall performance of the GMCs can be divided into three groups. In the first group (BCC-CSM2-MR, CESM2-WACCM), lower precipitation variability (STD <=1) than the measured data is observed. The moderate (STD>=2.5) precipitation variability (CMCC-CM2-SR5, CMCC-ESM2) over northern parts of the region is accompanied by relatively higher deviations in the south. The relatively highest (STD > 2.5) variability is seen in the other models that depict more intense precipitation variation than measured data during winter. BCC-CSM2-MR reported lower precipitation variability for the pre-monsoon season, whereas most GCMs (CESM-WACCM, CMSS-CM2-SR5, CMCC-ESM2, FGOALS-f3-L) reported moderate precipitation variability. The highest variability can be seen in EC-Earth3-AerChem, EC-Earth3-CC, MPI-ESM-1-2-HAM, and NorCPMI. For the monsoon season, moderate to highest precipitation variability depicts the GCM’s ability to report the occurrence of more intense precipitation than measured data with relatively higher deviations (STD =>2.5) in the eastern parts of the region. In the autumn season, the overall variability infers more dynamic precipitation in the model than observations with STD values above 3 mm/day. The ratio of STD infers that the spatiotemporal variability of the GCMs is higher than the observations implying more dynamics and intense precipitation in GCMs than measured data. The magnitude of STD is higher seasonally in regions with peak precipitation due to westerly and monsoon precipitation systems. The regional magnitude of the STD is relatively high in the north due to complex terrain; these results have also been acknowledged in a previous performance of remotely sensed and reanalysis data with similar differences [45].

**Fig 5:**
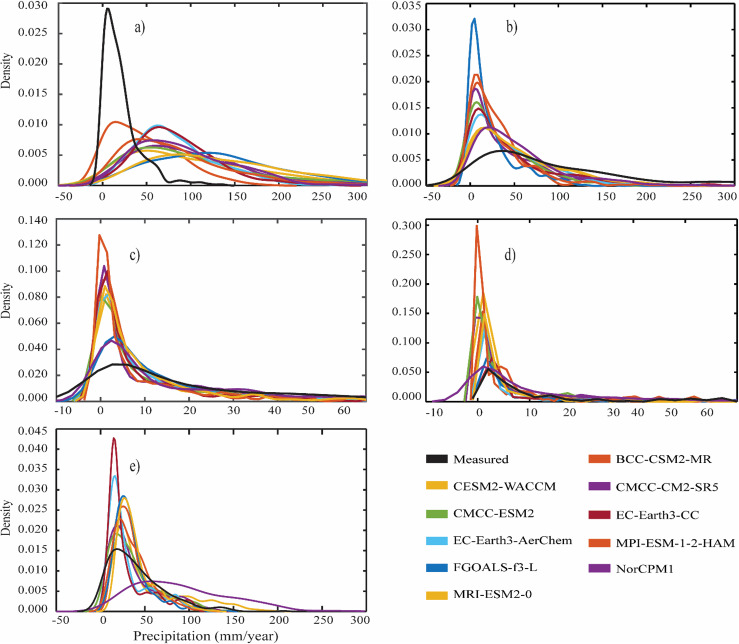
Probability density function (PDF) for annual-average precipitation during 1981–2014; **(a)** R1, **(b)** R2, **(c)** R3, **(d)** R4, and (e) whole country.

Fig 5 shows the probability density function (PDF) of the selected regions (a–d) and the whole country (e) averaged precipitation during 1981–2014. The descriptive statistics of the measured and GCMs precipitation moments of distribution are listed in Table 3, showing the precipitation variability parameters. For R1 (Fig 5a), the GCMs show a lower precipitation density in the range of <=50 mm and more in > 150 mm than the measured data. The heavy right-tailed distribution shows that the mean precipitation is largely driven by heavy precipitation with an overall lower asymmetry and kurtosis (Table 3). Considering the descriptive statistics, the relative spread is highest in CESM2-WACCM, EC-Earth3-CC, MRI-ESM2-0, and Nor-CPMI, with a moderate to higher spread than observed for most of the GCMs. In R2 (Fig 5b), the GCMs precipitation maximum density is located in the range of 0–100 mm concerning measured data. The descriptive statistics (Table 3) further show a relatively symmetrical distribution of the GCMs with higher skewness and kurtosis and lower deviations than measured data. In R3 (Fig 5c), the GCMs and measured data show a maximum precipitation density between 0–10 mm and a uniform spread of moderate precipitation. The low to moderate precipitation intensity (0–10 mm) events are more symmetrical and frequent than measured data, with a reasonable agreement for precipitation events ≥10 mm.

**Table 3:** Cumulative distribution of precipitation in the measured data and CMIP6 GCMs for regions (R1-R4), and the whole country.

Measured data and CMIP6 GCMs	Region 1			Region 2			Region 3			Region 4			Whole country		
Skewness	Kurtosis	STD	Skewness	Kurtosis	STD	Skewness	Kurtosis	STD	Skewness	Kurtosis	STD	Skewness	Kurtosis	STD
1. Measured data	8.38	110.14	2.40	5.09	46.49	7.52	7.88	93.55	3.94	12.44	203.39	4.16	1.35	5.31	32.04
2. BCC-CSM2-MR	3.14	16.34	2.91	5.78	56.63	2.33	11.14	166.57	1.74	15.57	353.27	1.65	1.55	5.23	23.44
3. CESM2-WACCM	4.22	35.39	6.74	9.21	147.91	5.25	16.85	431.17	2.55	15.63	373.61	3.55	1.22	3.49	41.81
4. CMCC-CM2-SR5	3.95	25.30	6.96	7.52	109.23	3.56	16.85	431.17	2.55	17.41	502.35	2.14	1.21	3.72	29.50
5. CMCC-ESM2	3.70	20.99	7.03	4.47	30.52	2.95	8.63	136.74	1.99	13.79	289.38	2.18	1.22	3.95	28.37
6. EC-Earth3-AerChem	3.07	16.25	4.70	6.04	59.84	4.10	8.03	91.50	1.93	9.55	120.98	2.55	1.39	4.12	24.94
7. EC-Earth3-CC	2.91	14.39	4.63	5.69	48.91	4.36	9.97	156.65	2.19	11.46	194.88	2.63	1.59	4.74	25.66
8. FGOALS-f3-L	3.77	25.31	8.31	12.40	225.64	3.98	2.31	10.06	1.33	16.45	329.88	3.99	1.76	7.01	20.36
9. MPI-ESM-1-2-HAM	3.36	18.01	4.99	8.20	102.37	3.58	6.20	52.58	1.59	13.60	248.57	0.93	1.12	4.17	21.20
10. MRI-ESM2-0	2.91	14.21	7.44	5.34	42.65	4.98	9.97	151.48	1.59	17.17	358.52	1.36	1.36	4.48	21.58
11. NorCPM1	2.95	15.24	4.90	4.70	34.91	4.29	6.44	63.29	2.50	6.48	59.02	2.52	0.74	3.16	53.61

For R4 (Fig 5d), the GCMs precipitation density is relatively higher than measured data for events of 0–10 mm, and moderate to heavy precipitation events have a similar density. The descriptive statistics (Table 3) further show relatively better symmetry of the GCMs and lower kurtosis than measured data, whereas the deviation and mean precipitation show higher variability in measured data than GCMs. The regional mean PDF (Fig 5e), shows that the maximum precipitation density falls at ~ 25 mm, as depicted by the measured data, followed by CMCC-CM2-SR5, CMCC-ESM2 EC-Earth3-AerChem, and EC-Earth3-CC. As shown by the skewness of the measured precipitation as a reference, the precipitation variability shows higher skewness (asymmetry) in the FGOALS-f3-L, EC-Earth3-CC, BCC-CSM2-MR, and moderate skewness (Table 3) in MRI-ESM2, indicating higher precipitation spread and magnitude than the measured data. The overall distributions of the CMIP6 GCMs show a significant move toward the right side, indicating more extreme precipitation events in the CMIP6 GCMs than in the measured data. The kurtosis and standard deviations of the CMIP6 GCMs show that the precipitation in those GCMs (FGOALS-f3-L, BCC-CSM2-MR, EC-Earth3-CC) is due to fewer/modest precipitation extremes when compared to the rest of the GCMs. The CMIP6 GCMs (except NorCPMI) and measured datasets exhibit a relative density of ≥0.010 to ≤0.045 for annual precipitation between 0–50 mm, inferring that all GCMs uniformly present maximum precipitation received an annual scale.

In conclusion, the CMIP6-GCMS can capture the precipitation density and reproduce the shape and pattern of the precipitation concerning the measured data. The distribution statistics indicate an overestimation of the precipitation magnitude; FGOALS-f3-L and NorCPMI exhibit the highest and lowest kurtosis and skewness compared with the measured data. Obvious overestimation of the moderate to low and uniform spread of the extreme precipitation events is evident, and hence bias correction may reduce these deviations before applications.

### 4.3. Seasonal and annual mean climatology

**Fig 6:**
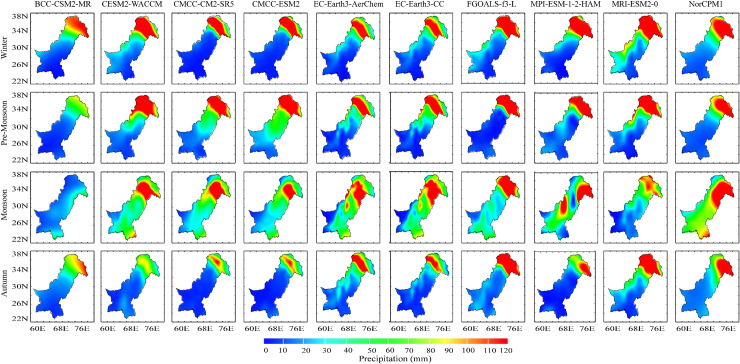
Climatological mean precipitation over Pakistan for winter, pre-monsoon, monsoon, and autumn seasons from the selected CMIP6 GCMs.

Fig 6 shows the seasonal mean precipitation climatology calculated from the CMIP6 data set for 1981–2014. The overall results show that all models exhibit a seasonal difference in precipitation. Significant spatial heterogeneity also exists in the intensity of precipitation over the study region. The spatial climatology of the GCMs shows a consistent precipitation pattern of the westerly precipitation systems in the north and northwestern regions of the country with peak precipitation and less in the south and southeast regions. Relatively consistent precipitation is observed in all CMIP 6 GCMs during the pre-monsoon season, with higher precipitation in the northern parts of the country and relatively lower precipitation in central, eastern, and western regions. Monsoon and autumn differences in precipitation follow the regional water cycle patterns in space and time in all the GCMs [58]. Furthermore, monsoonal precipitation follows a similar pattern with a relatively higher intensity of precipitation pattern at the northeast and southeast regions of the country. A maximum of ≥120 mm and ≤70 mm of precipitation are recorded in the country’s northern mountainous and southern coastal parts, respectively.

In terms of regional precipitation magnitude, R1 received the highest precipitation while R3 received the lowest precipitation amount both in the measured and GCMs data. The higher precipitation in R1 can be attributed to its distinct location and climate since this region is located in the northern mountains of the country, which are considered the intersection point of the westerly and monsoon weather systems. In addition, the northern parts (R1) of Pakistan are continuously experiencing convective weather processes in terms of light precipitation, which could add value to the total precipitation in winter, pre-monsoon, and monsoon seasons. On the other hand, R3 is comprised of the southwestern parts of Pakistan, which have an arid and hot climate with minimal influence of the two major precipitation systems, i.e., westerly and monsoon weather systems. It is worth mentioning that the southwestern parts of Pakistan are regularly affected by drought events in the recent past due to less precipitation and a hot climate. The drought episode of 1997–2002 is one the eye-witnessed, which caused substantial damages to the socio-economic conditions of the southwestern parts of the country.

**Fig 7:**
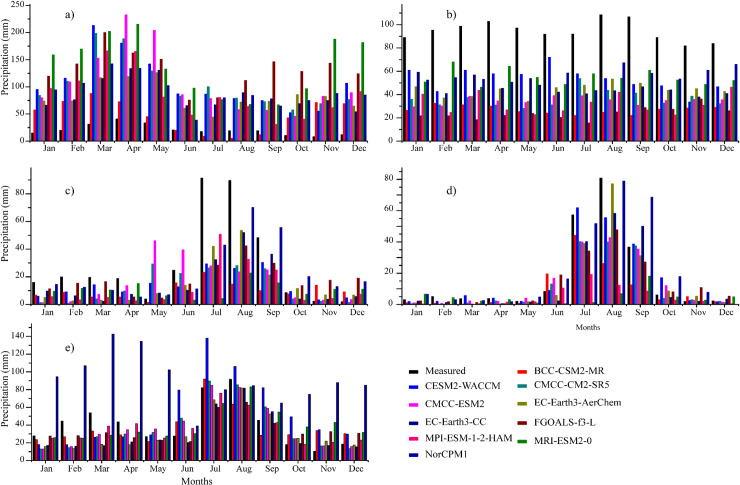
Climatological mean annual precipitation cycle at four selected locations and the whole country (Pakistan) **(a)** R1, **(b)** R2, **(c)** R3, **(d)** R4, and (e) whole country.

The mean annual precipitation cycle at four selected regions (Table 1) and the whole study area is shown in Fig 7a–d. The measured data shows that the maximum and minimum precipitation in R1 occurs during April (≥40 mm) and November (≤10 mm) (Fig 7a). Furthermore, all the CMIP6 GCMs overestimate the temporal climatology compared to the measured data (e.g., in the peak observed precipitation season nearly all models overestimate precipitation by about a factor of 3). Likewise, the measured data and all the GCMs perform correspondingly during the whole annual cycle; for instance, average monthly precipitation gradually increases during the first four months (Jan-April) with precipitation maxima (≥200 mm) under CMCC-ESM2 and MRI-ESM2-0. The mean precipitation from all the CMIP6 GCMs diverges for the rest of the year, showing an overestimated seasonality. Moreover, the BCC-CSM2-MR shows relatively better temporal climatology compared to the measured data for R1. Fig 7b shows the maximum precipitation during August (≥100 mm) and the minimum during November (~80 mm) in R2. Additionally, all the CMIP6 GCMs underestimate the temporal climatology (e.g., in the peak observed precipitation season is nearly ~ 100 mm and all models underestimate precipitation by about a factor of 0.60). Furthermore, CESM2-WACCM, MRI-ESM2-0, and NorCPM1 record relatively better seasonality of monthly average precipitation with a maximum of ≥70 mm and a minimum of ≥33 mm.

Fig 7c illustrates the precipitation pattern in R3, where the maximum precipitation is recorded during July and August (≥90 mm) and the minimum during November and December (≤5 mm). All the evaluated CMIP6 GCMs generally underestimate monthly average precipitation during the whole annual cycle and overestimate during May, November, and December, with the overall highest precipitation during July and August. Fig 7d depicts the seasonality of monthly precipitation for R4, where the maximum precipitation is observed during July, August, and September (35–80 mm). Like the measured data, a similar trend is observed in all the evaluated CMIP6 GCMs; however, all the models perform differently with a mixed trend over and underestimation during the whole annual cycle. Overall, the CESM2-WACCM model is found the best-performing model in R4. The climatological mean annual precipitation cycle of the whole country is presented in Fig 7e. According to measured precipitation, the maximum and minimum precipitation occurs during July-August (≥80 mm) and November (≤10 mm), respectively. CMCC-CM2-SR5 and CMCC-ESM2 show the closest agreement with the measured data during peak months (July-August) and lowest precipitating months (November). During winters, the lowest precipitation is reported during December (~18 mm) and increases gradually to the highest in March (~54mm), then declines in pre-monsoon during April, May, and June, and increases again abruptly during monsoon (July, August, September). CMCC-CM2-SR5, CMCC-ESM2, MRI-ESM2-0, and NorCPM1 exhibit closer agreement with the measured data during monsoon. CMCC-CM2-SR5, CMCC-ESM2, and EC-Earth3-CC illustrate the closest resemblance with the measured data during autumn. It should be further noted that NorCPM1 and CESM2-WACCM reported the highest precipitation during winter and summer, respectively.

In conclusion, both the measured data and CMIP6 GCMs have recorded relatively less precipitation during winter, while a large amount of precipitation is observed during summer in all regions. The regional-scale differences and deviations of precipitation regimes are mainly due to westerlies and South Asian Monsoon disturbances. In the country’s arid south and central regions, the GCM ability is much better for capturing precipitation’s absolute magnitude than in the northern terrains. The CMIP6 GCMs can simulate the regional precipitation spatiotemporal variability with relatively higher precipitation magnitude in the north that decreases towards the south. The decrease in precipitation magnitude from north to south parts can be attributed to their diverse climatic condition since northern and southern parts have humid and arid climates, respectively.

**Fig 8:**
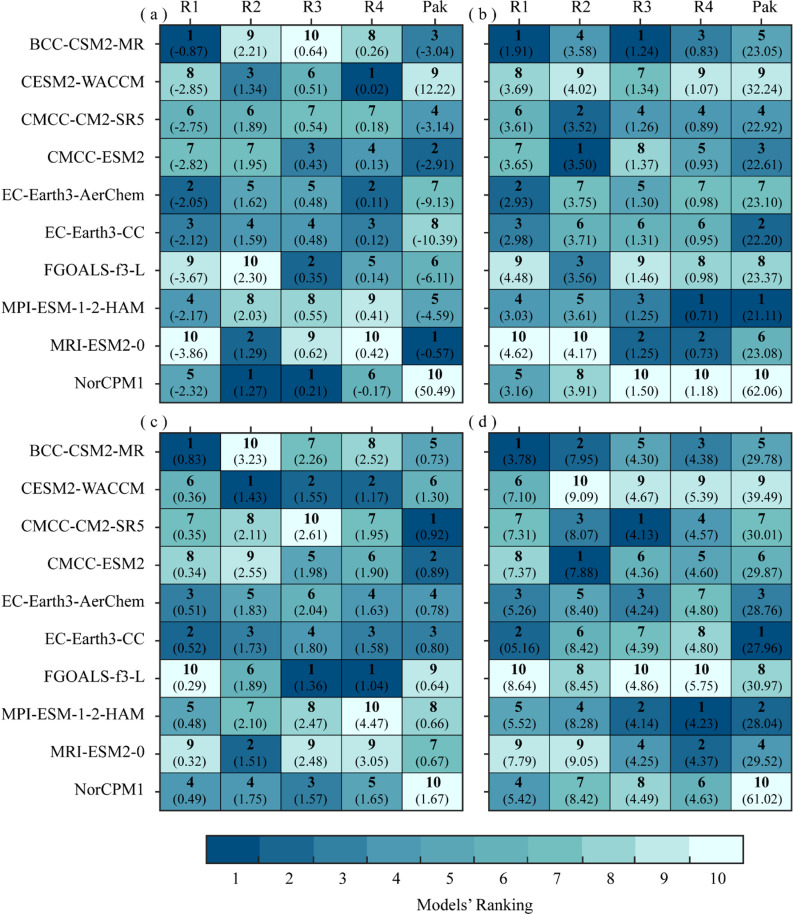
Portrait diagrams for models ranking in simulating daily precipitation over four regions and the whole country based on four error metrics; (a) bias, **(b)** MAD, **(c)** **STD, and** ***ub*****RMSD.** The bold values indicate the ranks of the selected models while the values in brackets indicate the actual values of each metric for the respective models.

Fig 8 shows the relative ranking of the selected GCMs based on individual error metrics, i.e., bias, MAD, STD, and *ub*RMSE at regional and country scales. Fig 8a shows the relative rankings of GCMs based on bias, BCC-CSM2-MR and MRI-ESM2-0 performed the best and worst in region 1 with bias values -0.87 and -2.32, respectively, while with slight overestimation NorCPM1 successfully reproduced the measured bias for region 2 and 3. CESM2-WACCM with the lowest bias of 0.02 performed the best in region 4. Furthermore, MRI-ESM2-0 with the lowest bias of -0.57 performed the best at the country level. In conclusion, all the models overestimate the measured data for R1 and underestimate measured data with the observed bias for R2, R3, and R4, except NorCPM1, which overestimates the measured value in R4. Except for R2, MRI-ESM2-0 performed the worst for all regions (1, 3, 4), but the best on the country scale. Fig 8b shows the relative rankings of GCMs based on MAD, BCC-CSM2-MR, CMCC-ESM2, BCC-CSM2-MR, and MPI-ESM-1-2-HAM exhibited the lowest MAD values (the best performance) for R1, R2, R3, and R4. MRI-ESM2-0 performed poorly in regions 1 and 2, while NorCPM1 found the worst for regions 3 and 4. In conclusion, all the GCMs possessed different amplitudes of mean absolute difference for different regions, MPI-ESM-1-2-HAM and NorCPM1 revealed the lowest/best and highest/worst amplitude at the country scale. Fig 8c unveiled the deviation of the ratio of amplitudes of GCMs from the measured data, BCC-CSM2-MR, CESM2-WACCM, FGOALS-f3-L, FGOALS-f3-L, and CMCC-CM2-SR5 possessed the closest deviations with measured data for R1, R2, R3, R4, and overall country. Similarly, the ubRMSD in Fig 8d represents the rankings based on unbiased differences in root mean squares of GCMs and measured datasets. BCC-CSM2-MR, CMCC-ESM2, CMCC-CM2-SR5, MPI-ESM-1-2-HAM, and EC-Earth3-CC exhibited the closest difference with observed data for R1, R2, R3, R4, and overall country, respectively, while FGOALS-f3-L has the largest difference for all regions and also for country scale.

The overall results of Fig 8 show that the BCC-CSM2-MR performed relatively well, followed by CESM2-WACCM, CMCC-CM2-SR5, CMCC-ESM2, and EC-Earth3-CC-AerChem, standing the best-performing models with respect to the measured records. In contrast, the performance of the MPI-ESM-1-2-HAM, MRI-ESM2-0, and NorCPM1 models was ranked the last, indicating their poor performance in reproducing precipitation extremes relative to the observed data.

**Fig 9:**
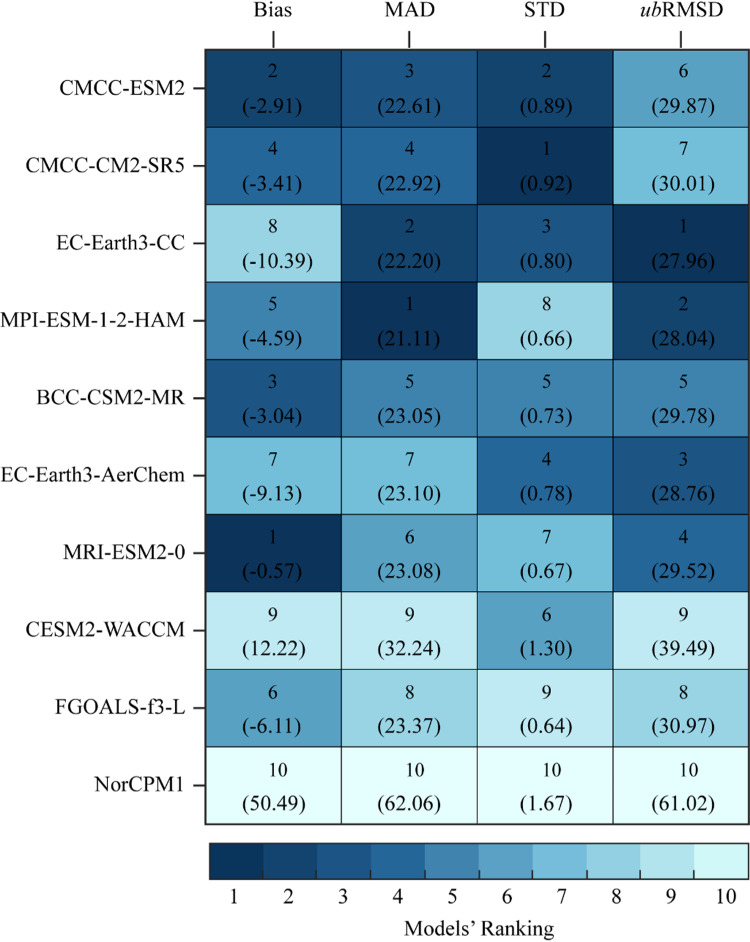
Relative ranking of GCMS in simulating daily precipitation by the rank of bias, MAD, STD, and *ub*RMSE metrics over the whole country (Pakistan).

In terms of bias, the highest and lowest overestimated bias with measured data is reported by EC-Earth3-CC (-10.39) and NorCPM1 (-0.17), and the underestimated bias ranges between 0.02 (CESM2-WACCM) -50.49 (NorCPM1). Considering the MAD values, except at the country scale (higher MAD values), all the models have shown the mean error within the range of 0.70–4.62, with the least errors in region 4. From the results of the normalized STD, it can be seen that the selected models displayed deviations and amplitudes in the range of 0.29–4.47, against the observed data. Since the STD value closer to 1 is the indication of an optimum model, thus, the CMCC-CM2-SR5 is considered the best-performing model at the country scale with an STD value of 0.92, followed by CMCC-ESM2 (0.89) and EC-Earth3-CC (0.80). Similarly, the ubRMSE showed that most of the selected models reproduced the error differences within the range of 3.78 - 61.02, with the lowest differences by BCC-CSM2-MR in R1, followed by CMCC-CM2-SR5 in R3. The least error differences of these models indicate a relatively close agreement with the measured data. On the contrary, the NorCPM1 has shown the highest error differences at the rate of 61.02 at the country scale, suggesting its poor performance in the simulation of historical precipitation extremes over Pakistan. Furthermore, Fig 9 depicts the overall ranking of CMIP6 GCMs based on selected metrics (collective), CMCC-ESM2 outperformed in simulating historical precipitation over Pakistan, followed by CMCC-CM2-CR5 and EC-Earth-CC, NorCPM1 is found to be the most incompatible with measure data in reproducing closer agreement with Bias, MAE, STD and *ub*RMSD.

## 5. Discussion

The comparison in this research involves ten CMIP6 GCMs and the observation of station precipitation data from 1981 to 2014 in various climatic zones of Pakistan. This comparison is conducted across daily, seasonal, interannual, and annual time scales [45,58]. The daily variability exhibits that all the GCMs skillfully reproduced precipitation frequency following the measured data in the semiarid region, with underestimations in humid, arid, and extremely arid regions. The daily scale variability shows an overestimation of precipitating days in northern parts of Pakistan with an underestimation in southern Pakistan in all GCMs. Furthermore, the intermodal spread of the frequency and intensity regionally varied with relatively smaller deviations in the north and larger deviations in the southern parts, inferring differences in precipitating days with more events in the north than the south. The GCMs demonstrate strong performance in capturing events with precipitation of ≥10 mm/day, as evidenced by the frequency and intensity of different bins, which supports previous research findings [59–62]. The disagreement (over/underestimation) of GCMs across each region is moderate to low for events ≤ 10 mm/day and a relatively higher divergence is evident for events ≥10 mm/day, which was also reported by Liu et al. [63] and, Assamnew and Tsidu [64].

The seasonal precipitation magnitude is highly variable across Pakistan [65,66] along latitude and seasons. The ratio of standard deviation shows higher precipitation variation in the GCMs than observed precipitation data. The low to moderate variability is seen in CESM-WACCM, CMSS-CM2-SR5, CMCC-ESM2, and FGOALS-f3-L models during lower precipitating seasons. The GCMs’ deviation ratio indicates an increase in precipitation extremes and intensities, along with the familiar terrain effects in the north, consistent with findings from reanalysis and remotely sensed precipitation data [45]. A suite of bias correction and downscaling techniques can suffice for such biases in magnitude knowing the implications of the bias correction and downscaling techniques beforehand [67]. The overestimation of precipitation, biases, and increased occurrence of extreme precipitation events have been previously documented in CMIP6 CGMs and observations across various regions worldwide [67,68]. The IPCC AR6 stated that the occurrence of heavy precipitation events will intensify and become more frequent in most regions under different global warming targets (IPCC 2022) [69].

The possible reasons for the inter-model spread, deviations, over and underestimation, and wet and dry biases in the north and south of Pakistan could be due to different degrees of sensitivity of regional precipitation to natural and anthropogenic forcing compared to CMIP5 [1,16,67,68,70]. The regional probability density function (PDF) of the GCMs relative to the observation shows a higher density of the lower to moderate precipitation events and heavy-tailed deviations with lower kurtosis and asymmetry. The country’s mean precipitation pattern is relatively symmetrical with observations except for NorCPM1 with maximum skewness. The rest of the GCMs show the maximum density of precipitation events in close agreement with observations. The GCMs show intermodal deviations and variability for different precipitation magnitudes and densities, but the overall distribution is preserved reasonably and can be further improved with bias correction and downscaling. The observed mean precipitation has increased over most of the tropical areas and high latitudes while a decrease has been found in most sub-tropical areas [69]. Using the bias, MAD, STD, and *ub*RMSD, the best-performing models identified include BCC-CSM2-MR, followed by CESM2-WACCM, CMCC-CM2-SR5, CMCC-ESM2, and EC-Earth3-CC-AerChem, the performance of the MPI-ESM-1-2-HAM, MRI-ESM2-0, and NorCPM1 models ranked last, indicating their poor performance in reproducing precipitation extremes relative to the observed data.

Similarly, the HadGEM3-GC31-MM model demonstrates the highest skill in simulating precipitation distributions, followed by several other models like EC-Earth3-Veg-LR and CNRM-ESM2-1 [71]. These models project a substantial increase in precipitation under high emission scenarios (SSP3-7.0 and SSP5-8.5) and a modest rise under low emission scenarios (SSP1-2.6 and SSP2-4.5) for Pakistan [71]. Ali et al. [72], revealed that CMIP6 models generally project increased precipitation for Pakistan, while EC-Earth3-Veg, MRI-ESM2-0 and Nor-ESM2-MM were found to be the best models in replicating historical and simulating future precipitation extremes over Pakistan. Ali et al. [70], indicate a slight decrease in precipitation under the RCP8.5 scenario for Pakistan as a whole, while projecting an increase for the Upper Indus Basin region. This highlights the spatial variability in precipitation projections within the country [73]. CMIP6 models offer enhanced precipitation simulations over Pakistan, with most projections indicating an increase in future precipitation, especially under high emission scenarios [74]. The findings from CMIP6 models are crucial for water resource management, food security, and disaster risk management in Pakistan [71,72].

The regional (spatial) and annual (temporal) precipitation pattern highlights that the precipitation cycle of the study area exhibits latitudinal and longitudinal variability decreasing from north to south and increasing from west to east during monsoon and pre-monsoon, respectively. The GCMs show lower regional biases in arid and plain areas compared to the complex terrains in the northern regions, which is consistent with findings from earlier research [75]. The varied topography of Pakistan contains flat plains in the east, west, and southern parts of the country, with the complex terrain of the Karakoram range in the northern parts possessing uncertain precipitation patterns [76]. The variability of precipitation shifting patterns may also be associated with the variations in atmospheric circulations in the country [7,22]. Recent studies have also reported a diverse shifting trend in precipitation extremes [7,70]. The complex terrain-induced deviations, precipitation magnitudes, measured data uncertainties, and low gauge network density cause regional differences in the performance of GCMs [58,77,78]. Therefore, the performance of GCMs may also vary with the regional and climatic distribution of the study area [26]. Thus, the interpretation of the results should consider and acknowledge these aspects when using the CMIP6 GCMs for regional-scale applications.

## 6. Conclusions

This study assessed the performance of ten CMIP6 GCMs at daily, seasonal, interannual, and annual scales in simulating the historical precipitation over Pakistan. At a daily scale, the frequency of precipitating days ≤10 mm/day is well captured in all regions, with deviation for days > 10 mm/day precipitation. As the precipitation intensity increases, model performance skills in capturing higher intensity also increase. The precipitation’s seasonal deviation shows the model’s ability to report a more dynamic precipitation profile than observations. The GCMs can show higher densities of lower to moderate precipitation but report more extreme precipitation events than the observed precipitation. In general, the semiarid and arid regions can be better captured by the CMIP6 GCMs. This study could benchmark and recommend suitable GCMs for diversified regions with different climates and complex terrains. The differences and variations need further exploration with potential reasons, and detailed studies should be performed in different regions at different time scales.
